# Cost-effectiveness of 3-months isoniazid and rifapentine compared to 9-months isoniazid for latent tuberculosis infection: a systematic review

**DOI:** 10.1186/s12889-022-14766-6

**Published:** 2022-12-07

**Authors:** Wendy A. Lai, Kaitlyn Brethour, Olivia D’Silva, Richard E. Chaisson, Alice A. Zwerling

**Affiliations:** 1 University of Toronto Department of Family and Community Medicine, Toronto, Canada; 2 University of Ottawa Department of Chemistry and Biomolecular Science, Ottawa, Canada; 3 University of Ottawa School of Epidemiology and Public Health, Ottawa, Canada; 4 Johns Hopkins Center for Tuberculosis Research, Baltimore, USA

**Keywords:** Tuberculosis, Preventive treatment, Rifapentine, Isoniazid, Systematic review, Cost-effectiveness

## Abstract

**Background:**

We conducted a systematic review examining the cost effectiveness of a 3-month course of isoniazid and rifapentine, known as 3HP, given by directly observed treatment, compared to 9 months of isoniazid that is directly observed or self-administered, for latent tuberculosis infection. 3HP has shown to be effective in reducing progression to active tuberculosis and like other short-course regimens, has higher treatment completion rates compared to standard regimens such as 9 months of isoniazid. Decision makers would benefit from knowing if the higher up-front costs of rifapentine and of the human resources needed for directly observed treatment are worth the investment for improved outcomes.

**Methods:**

We searched PubMed, Embase, CINAHL, LILACS, and Web of Science up to February 2022 with search concepts combining latent tuberculosis infection, directly observed treatment, and cost or cost-effectiveness. Studies included were in English or French, on human subjects, with latent tuberculosis infection, provided information on specified anti-tubercular therapy regimens, had a directly observed treatment arm, and described outcomes with some cost or economic data. We excluded posters and abstracts, treatment for multiple drug resistant tuberculosis, and combined testing and treatment strategies. We then restricted our findings to studies examining directly-observed 3HP for comparison. The primary outcome was the cost and cost-effectiveness of directly-observed 3HP.

**Results:**

We identified 3 costing studies and 7 cost-effectiveness studies. The 3 costing studies compared directly-observed 3HP to directly-observed 9 months of isoniazid. Of the 7 cost-effectiveness studies, 4 were modelling studies based in high-income countries; one study was modelled on a high tuberculosis incidence population in the Canadian Arctic, using empiric costing data from that setting; and 2 studies were conducted in a low-income, high HIV-coinfection rate population. In five studies, directly-observed 3HP compared to self-administered isoniazid for 9 months in high-income countries, has incremental cost-effectiveness ratios that range from cost-saving to $5418 USD/QALY gained. While limited, existing evidence suggests 3HP may not be cost-effective in low-income, high HIV-coinfection settings.

**Conclusion:**

Cost-effectiveness should continue to be assessed for programmatic planning and scale-up, and may vary depending on existing systems and local context, including prevalence rates and patient expectations and preferences.

**Supplementary Information:**

The online version contains supplementary material available at 10.1186/s12889-022-14766-6.

## Introduction

One-quarter of the world has latent tuberculosis infection (LTBI); an estimated 10% will eventually develop active tuberculosis (TB) [1]. This LTBI population functions as the reservoir driving ongoing incidence of active TB, even in the absence of continued transmission. Finding and treating LTBI is important to control TB and for the ultimate goal of eliminating TB [1–3].

For many years, LTBI has been treated with isoniazid administered daily for 9 months (9H), which reduces reactivation by 90% but incurs risk of hepatoxicity [4, 5]. Isoniazid is widely-available, and costs very little.

More recently, shorter options for LTBI treatment have been adopted [6], including a three-month regimen of once-weekly isoniazid and rifapentine (3HP) [7]; 4 months of daily rifampicin (4R) [8]; or 3 months of daily isoniazid and rifampicin [6]. Shorter courses of treatment are similarly effective and easier for patients to complete [9]. 4R is associated with a theoretical risk of rifampicin resistance, though not empirically demonstrated [10]. 3HP is effective [7] but is usually given via directly-observed treatment (DOT), which requires more visits and human resources, and has corresponding budgetary implications. Self-administration of 3HP met the threshold for non-inferiority to DOT in [11], and is an approved strategy for, the US [12]. However, rifapentine is significantly more expensive than isoniazid.

Directly-observed preventative therapy (DOPT) for treatment of LTBI is a commonly used programmatic strategy, particularly in populations at high-risk of developing active TB [13–17]. This treatment support might mitigate the risk related to congregate or crowded living [14, 15], cultural and/or linguistic barriers [13, 14]. Barriers such as lack of trust in authorities, and histories of racism and colonialism may pose challenges for DOPT [18, 19]. While DOPT can be used with any LTBI regimen, it is recommended with 3HP because of the weekly dosing interval and significant pharmacokinetic impact of a missed dose.

A 2011 systematic review evaluated cost effectiveness evidence for LTBI treatment overall [20]. It excluded high-risk populations including HIV-coinfected patients, but did not restrict drug regimens, including self-administered regimens and DOT. Isoniazid was cost-effective for the general population in high-resource settings compared to no treatment, but there was insufficient evidence for low-resource settings. The systematic review included only one study directly comparing two drug regimens.

Since 2011, several more cost-effectiveness studies have been published. With 3HP, the requirement for direct observation coupled with expensive medication incurs more up-front costs and could be cause for policymakers to hesitate. Economic evidence to inform these decisions is critical.

We performed a systematic review on the cost-effectiveness of 3HP, recognizing potential variation across settings, geographic regions, and specific populations.

## Methods

A systematic review was performed to determine the cost and cost-effectiveness of 3HP DOT. We searched PubMed, Embase, CINAHL, LILACS, and Web of Science up to February 28, 2022, with search concepts combining latent tuberculosis, drug therapy or directly observed preventive therapy, and cost, economic, or cost effectiveness (see Additional file 1 for full details on the search strategy).

Studies included were in English or French, on human subjects, with LTBI, provided information on specified anti-tubercular regimens, had a directly-observed arm, and described outcomes with some cost or economic data. We excluded posters and abstracts, studies on drug-resistant TB, and studies that combine testing and treating as a single intervention. Two reviewers (KB, WAL) screened all records independently; disagreements were resolved by consensus and if necessary by a third reviewer (AAZ). We included only studies examining 3HP DOT cost or cost-effectiveness. Data extraction was based on modified criteria from CHEERS checklist [21]. The eligible studies were extracted independently by two reviewers (KB, WAL).

Where there was no description of whether treatment was self-administered therapy (SAT) or administered by DOT, we assumed the usual strategy of administration based on drug regimen (e.g. 3HP was originally studied as DOT [7], 9H is usually self-administered) or based on national standards (e.g. LTBI treatment in Taiwan is routinely given by DOT) or by inference from information given regarding the frequency of visits or dosing.

We included studies that report data on cost with no information or follow-up with regard to effectiveness. We report on cost studies and cost-effectiveness studies separately, with outcomes on cost per patient and incremental cost effectiveness ratios (ICERs), respectively. For cases when authors provided itemized costs, such as for medications, medical professionals’ time, and laboratory and radiology investigations, we calculated estimated total costs per regimen for comparison. Quality of the included studies for the cost and cost-effectiveness review was assessed independently by two reviewers (KB, WAL) based on a modified Drummond checklist [22]. We did not plan for a meta-analysis given the range of geographical and contextual factors, including baseline adherence rate, LTBI prevalence, co-morbidity prevalence particularly HIV co-infection, usual care for LTBI treatment, and patient expectations.

For cost-effectiveness studies, we present results on 3HP DOT and 9H SAT. For studies in which there was no 9H SAT arm, we compared 3HP DOT to the comparator most similar to 9H SAT for which data was available (e.g. 9H DOT). ICERs were re-calculated using 9H SAT as baseline where possible. Costs have been converted to 2020USD based on Consumer Price Index [23].

Cost and cost-effectiveness analysis studies on self-administered 3HP (3HP SAT) alone, with no DOT arm, were excluded from the formal systematic review. Our primary outcome is cost and cost-effectiveness for 3HP.

## Results

**Fig. 1:**
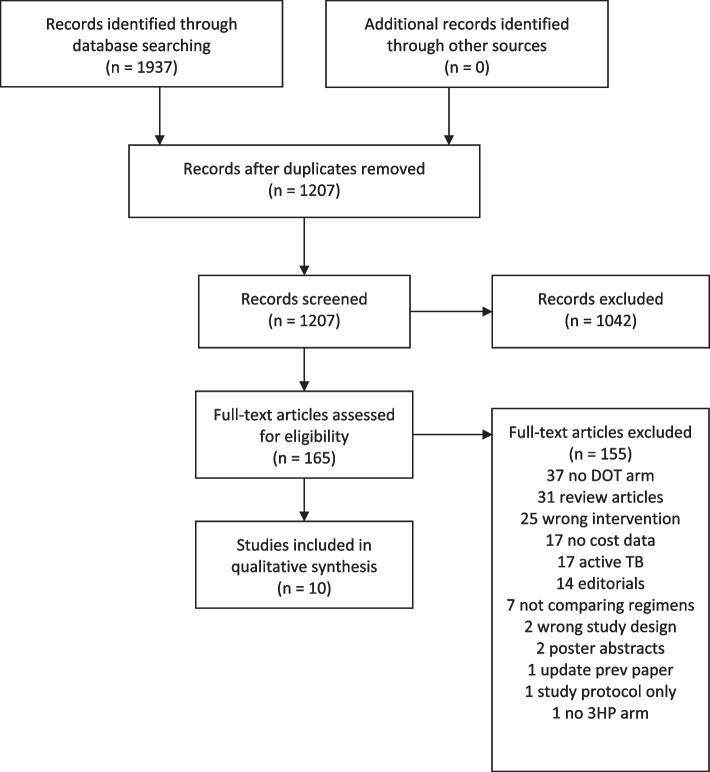
PRISMA 2009 Flow Diagram. Directly-observed 3HP cost and cost-effectiveness systematic review [24]

We identified 1937 records from database searches, 730 were duplicates, leaving 1207 for screening. Of these, 165 underwent full text review. Ten papers were included in this systematic review (see Fig. 1). We identified three costing-only studies and seven cost-effectiveness studies. Key study parameters are shown in Table 1. (See Additional file 2 for full details.)

**Table 1:** Study characteristics

	Author, year	Population, context, setting	study perspective	reference case	comparators	time horizon	Primary outcome	cost vs CEA^c^	Secondary/ health outcomes	WTP threshold	source of cost data	source of effectiveness data	currency, price date	choice of model
Costing studies	Huang 2016 [25]	general population^a^, Taiwan, hospital	health system	9H DOT	3HP DOT	5 years	cost/ patient	cost	treatment completion, active TB, cost/case avoided	NA	empiric	published data	USD^d^2014	NA
	Chen 2018 [26]	rheumatoid arthritis patients, Taiwan, clinic	health system	9H DOT	3HP DOT	2 years	cost/ patient	cost	treatment completion, active TB, cost/case avoided	NA	empiric	NA	USD^d^2014	NA
	Wheeler 2019 [27]	Inmates, USA, prison clinic	health system	9H DOT	3HP DOT	no long-term follow-up	cost/ patient	cost	treatment completion	NA	empiric	NA	USD 2012	NA
Cost effectiveness studies (HIC context)	Holland 2009 [28]	general population, USA, NS^b^	societal	no treatment	9H SAT, 9H DOT, 3HP DOT, 4R	lifetime	ICER	CEA	active TB	$50,000	mixed	mostly published	USD 2008	Markov
	Holland 2011 [29]	general population, USA, NS^b^	societal	9H SAT	3HP SAT, 3HP DOT, 1HP SAT	lifetime	ICER	CEA	active TB	0	non-empiric	published	USD 2011	Markov
	Shepardson 2013 [30]	general population, USA, clinic	health system and societal	9H SAT	3HP DOT	20 years	ICER	CEA	active TB	NS^b^	mixed	published data	USD 2010	individual-based stochastic
	Doan 2019 [31]	general population, USA, NS^b^	health system	serial radiographic surveillance	3HP DOT, 3HP SAT, 3RH, 4R, 6H, 9H	20 years or death	ICER	CEA	active TB	$50,000	non-empiric	published data	USD 2018	Markov
	Pease, 2021 [32]	general population, Iqaluit, Canada	health system	9H DOT (twice weekly)	3HP DOT	30 years	ICER	CEA	Cases and deaths averted	NS^b^	mostly empiric	programmatic	CAD 2019	Markov
Cost effectiveness studies (LMIC context)	Johnson 2018 [33]	people living with HIV, Uganda, HIV clinic	health system	9H SAT	3HP-DOT	20 years	ICER	CEA	active TB	sensitivity-tested $1000, $3000, $5000, $7000, $9000	non-empiric	published data	USD 2017	Markov
	Ferguson, 2020 [34]	people living with HIV, Uganda, HIV clinic	health system	3HP DOT	1HP SAT	20 years	ICER	CEA	Active TB, TB deaths	NS^b^	Non-empiric	Published data	USD 2019	Markov

^a^but no women of child-bearing age, no children < 12 years old

^b^*NS* not stated

^c^CEA = cost-effectiveness analysis

^d^denotes currency year not explicitly stated, so year inferred for subsequent CPI calculation

### Costing-only studies

The three costing-only studies compare treatments that are directly observed; none has a SAT group. Two are from hospitals in Taiwan [25, 26], where DOPT is the standard of care. The other pertains to Californian prisoners [27]. All 3 costing studies reported completion rates, and consistently reported improved completion rates [25–27] for 3HP compared to 9H (See Table 2).

**Table 2:** Cost/patient, as found in costing studies and compared to input costs/patient in cost-effectiveness studies, adjusted to 2020 USD, and completion rates

		Study type	Cost/ patient 9H DOT	Cost/ patient 9H SAT	Cost/ patient 3HP DOT	Treatment completion 9H DOT	Treatment completion 9H SAT	Treatment completion 3HP DOT
Costing studies	Huang [25]	Costing	$784		$286	0.873		0.97
	Chen [26]	Costing	$784		$286	0.783		0.905
	Wheeler [27]	Costing	$636		$676	0.42		0.9
Cost effectiveness studies (HIC)	Holland 2009 [28]	CE		$285	$605		0.53	0.94
	Holland 2011 [29]	CE		$287	$609		0.53	0.9
	Shepardson [30]	CE		$479	$691		0.68	0.84
	Doan [31]	CE		$496	$619		0.52	0.85
	Pease [32]	CE	$801		$381	0.75		0.82
Cost effectiveness studies (LMIC context)	Johnson [33]	CE		$17	$94		0.47	0.74
	Ferguson [34]	CE			$23			0.74

Costs are rounded to nearest whole dollar and adjusted to 2020 USD using Consumer Price Index [23]

All three costing studies are in contexts that have systems in place for DOT, so no new investments had to be made. With shorter duration and longer dosing interval, 3HP is favoured because of fewer total visits.

Treatment completion rates vary widely between studies, particularly for 9H (see Table 2). This heterogeneity may reflect regional or cultural differences in treatment acceptance and adherence.

Costing studies did not include any downstream costs of LTBI treatment, notably for serious adverse events (SAEs) and for active tuberculosis. Therefore, they had fewer component costs compared to the cost-analysis studies (see Table 3).

**Table 3:** Items included in cost inputs for cost-effectiveness and cost studies

	Author	TB meds	physician time	nurse time	other worker time	“clinic visit”	travel	radiology	lab tests	patient time (lost wages)	treatment SAEs	hospitalization SAE	treatment active TB	hospitalization active TB	contact tracing	HIV meds	secondary transmission
Costing studies	Huang [25]	Y			Y												
	Chen [26]	Y			Y												
	Wheeler [27]	Y	Y	Y	Y			Y	Y								
Cost effectiveness studies	Holland 2009 [28]	Y	Y	Y	Y		Y		Y	Y	Y	Y	Y	Y	Y		Y
	Holland 2011 [29]	Y	Y	Y	Y		Y		Y	Y	Y	Y	Y	Y	Y		Y
	Shepardson 2013 [30]	Y	Y	Y	Y	Y		Y	Y	Y	Y	Y	Y	Y			Y
	Doan [31]	Y	Y		Y			Y	Y		Y	Y	Y	Y			
	Pease [32]	Y	Y	Y				Y	Y		Y	Y	Y	Y			
LMIC context	Johnson [33]	Y				Y							Y			Y	
	Ferguson [34]	Y				Y							Y			Y	

### Cost-effectiveness studies

Of seven identified cost-effectiveness studies, four are modelled on US data, a high-resource setting [28–31]. Pease [32] focuses on the Canadian Arctic that is high income, high TB prevalence, geographically remote, and a majority-Indigenous population. Johnson [33] and Ferguson [34] examine cost-effectiveness in a low-resource, high HIV prevalence setting, using a Ugandan HIV clinic as their model, with different reactivation rate, mortality rate, treatment standards, and willingness-to-pay (WTP) (Table 4 and Additional file 3) compared with North America.

**Table 4:** Selected epidemiologic and cost input parameters in 2020 USD used across included studies

Author	Holland 2009 [28]	Holland 2011 [29]	Shepardson [30]	Doan [31]	Pease [32]	Johnson [33]	Ferguson [34]
*Selected epidemiologic parameters*							
Risk reduction 9H SAT	0.93	0.93	0.92	0.96	0.93^b^	0.58	na
Risk reduction 3HP DOT	0.93	0.93	0.95	0.975	0.93	0.63	0.90
Treatment completion 9H SAT	0.53	0.53	0.68	0.52	0.75^b^	0.47	na
Treatment completion 3HP DOT	0.94	0.9	0.84	0.85	0.82	0.74	0.74
Adverse events 9H SAT	0.014	0.014	0.055	0.023	0.065^b^	0.034	na
Adverse events 3HP DOT	0.014	0.05	0.082	0.016	0.057	0.034	0.034
*Selected cost parameters*^*a*^							
Treatment cost per regimen 9H SAT	285	287	479	496	801^b^	18	na
Treatment cost per regimen 3HP DOT	605	609	691	619	381	94	23
Treatment cost of SAEs	190	190	201	Included in cost of hospitalization	Included in cost of hospitalization	Not included	Not included
Cost of hospitalization SAEs	6396	6372	6738	6670	2616	Not included	Not included
Cost active TB meds	2460	2426	3542	3812	1517	230	230
Cost active TB hospitalization	12,016	11,969	30,260	29,463	66,495	Not included	Not included

*na* not applicable. “Not included” denotes not included in the model. SAEs = serious adverse events

^a^Costs in 2020 USD after adjustment using Consumer Price Index [23]

^b^regimen was 9H DOT

The five cost-effectiveness studies in high-income settings vary on whether 3HP or 9H is better tolerated (Table 4). Pease [32] is unique with less difference in treatment completion between 3HP and 9H DOT, with higher 9H treatment cost (since it was DOT rather than SAT); and lower 3HP DOT treatment cost. Additionally, the cost of treating active TB is higher, likely due to geographical remoteness.

Five cost-effectiveness studies in high-income settings demonstrate widely-variable ICERs ranging from 3HP being cost-saving compared to 9H, to an ICER of $5418 per QALY gained, though all found that 3HP DOT is cost-effective with ICER values well under a WTP of USD $50,000 per QALY gained. Methods for the five were comparable, with a few inclusion differences such as contact tracing, secondary transmission, travel times, patient costs, and radiology (Table 3). All seven cost-effectiveness studies list one or more clinical outcomes; active TB cases per 1000 patients is common to all, ranging from 9.1 to 37 per 1000 patients treated with 9H, and 3.9 to 38 per 1000 patients treated with 3HP (Table 5).

**Table 5:** Outcomes, adjusted to 2020 USD

	Cost/ patient 9H SAT	Cost/ patient 3HP DOT	Effectiveness (QALY gained/patient 9H SAT)	Effectiveness (QALY gained/patient 3HP DOT)	ICER 3HP DOT compared to 9H SAT (2020USD/QALY gained)	Total TB cases/ 1000 patients on 9H SAT	Total TB cases/ 1000 patients on 3HP DOT
Holland 2009 [28]	$817	$933	22.64505	22.67083	^a^4511	20.3	8.7
Holland 2011 [29]	$833	$868	22.64937	22.66836	^a^1818	22	13
Shepardson 2013—health system [30]	$606	$739	0.044^d^	0.019d	5418	9.1	3.9
Shepardson 2013—societal [30]	$837	$864	0.044^d^	0.019^d^	1081	9.1	3.9
Shepardson 2014—health system [35]	Not stated	Not stated	Not stated	Not stated	2054	9.1	3.9
Shepardson 2014—societal [35]	Not stated	Not stated	Not stated	Not stated	3HP dominates	9.1	3.9
Doan [31]	$1095	$900	15.6161	15.6539	^a^3HP dominates	23.2	10.6
Pease [32]	$920^c^	$626	20.13^c^	20.14	3HP dominates	30.16	27.79
Johnson [33]	$2576	$2640	10.843	10.837	^b^9927 DALY averted	37	28
Ferguson [34]	Not studied	$1541	Not studied	7.3697	Not applicable	Not studied	21.3

Costs in 2020 USD after adjustment using Consumer Price Index [23]

^a^ICERs recalculated using 9H SAT as reference

^b^2020USD/ DALY averted in a low-resource setting

^c^9H DOT was reference

^d^Shepardson reports effectiveness as mean QALY loss

All authors report from a health system perspective, except for Shepardson who provides two analyses as denoted

Pease [32] compares 9H DOT against 3HP DOT in a high-incidence, high-income, remote Arctic setting and finds that 3HP DOT is cost-saving in almost all scenarios. Shepardson in a US setting makes analyses from health system and societal perspectives [30], demonstrating 3HP DOT’s ICER of USD $1081 per QALY gained from a societal perspective (Table 5). After a rifapentine cost-reduction, Shepardson recalculated the ICER and found 3HP DOT to be cost-saving compared to 9H SAT from a societal perspective [35]. Note that Shepardson reports effectiveness as mean QALY loss, rather than QALY gained, though ICERs are calculated as incremental cost per QALY gained (see Table 5).

Holland 2009 also directly compares 9H DOT to 9H SAT and found it *not* to be cost-effective, with an ICER well over USD $50,000 per QALY gained [28].

The study by Johnson, in a low-resource, high-prevalence setting with high HIV co-morbidity omits many costs, such as for treating adverse events or for hospitalization, while HIV treatment costs are included. This leads to higher overall cost per DALY averted [33]. Completion rates for both regimens are lower than other studies, particularly for 3HP DOT (see Table 4). Risk reductions on both regimens are lower, contributing to a higher ICER. Risk of adverse events, including drug-drug interactions, were assumed to be equivalent. Johnson concludes that in a low-resource setting, ICER is above a WTP of USD$1000/DALY averted (Table 5).

Based on a similar model, Ferguson [34] performed a cost-effectiveness analysis for 1HP compared to 3HP. 1HP is administered daily by self-administration, in contrast to 3HP which is administered weekly by DOT. There is no isoniazid arm and regimens are presumed equally effective [36], so this is not comparable to other studies and there is no inherent ICER. Compared to the Johnson study [33], input costs and overall costs are lower for 3HP DOT.

For high-resource settings, the ICER is expressed as dollars per Quality-Adjusted Life Year (QALY) gained but for a low-resource setting, the ICER is expressed as dollars per Disability-Adjusted Life Year (DALY) averted as per convention, and so these are not comparable.

The quality of the included studies, assessed on a modified Drummond checklist [22], is provided in Additional file 4. Of note, the costing studies [25–27] do not include all the costs incurred, particularly long-term, downstream costs/savings. They also do not include sensitivity analyses. Modelling studies in HICs by Doan [31], Holland [28, 29], and Shepardson [30] have no discussion related to ethical and distribution issues, though Pease [32] includes this. The modelling studies set in LMICs [33, 34] do not include the cost of adverse events. Chen [26] and Huang [25], who study Taiwanese patients, do not address generalizability of their findings.

## Discussion and conclusion

This is the first systematic review on the cost-effectiveness of 3HP compared to 9H for LTBI. All studies in highly-resourced contexts found that 3HP DOT is cost-effective at a WTP of $50,000 per QALY gained, compared to 9H SAT or 9H DOT, despite the higher cost of rifapentine and the costs of DOT. Doan and Pease found it to be cost-saving [31, 32]. Shepardson’s 2014 update found 3HP to be cost-saving from a societal perspective [35]. However, in LMICs, 3HP DOT may not be cost effective. Johnson [33] found 3HP to have an ICER above a WTP of USD$1000/DALY averted. Ferguson did not compare 3HP to 9H [34].

Self-administered treatment studies were specifically excluded from this systematic review. Because missing a dose in a weekly regimen can lead to subtherapeutic drug levels, treatment failure, and development of drug resistance, we focus on 3HP DOT as it is habitually administered and studied. In a non-inferiority trial, 3HP SAT was found to be non-inferior to 3HP DOT in the US [11]. Scant literature examines 3HP SAT cost and cost-effectiveness. Denholm [37] demonstrated in a single Australian centre that 3HP SAT costs less than 9H SAT, at $375 compared to $441 USD per person treated, driven by more outpatient visits in the 9H arm. Yuen [38] examined costs of 3HP SAT compared to 6H SAT in Pakistan, demonstrating that in this LMIC, 3HP is also less costly, particularly after a rifapentine price reduction which resulted in 3HP SAT costing $294 USD compared to $399 for 6H SAT. Holland 2011 and Doan included a 3HP SAT regimen in their models: both studies found it more cost-effective than 3HP DOT and 9H SAT. [29, 31]

The heterogeneity found in the costing and cost-effectiveness studies might be in part because of qualitative aspects to how DOPT is operationalized. The literature supports measures that improve the ease of LTBI treatment, such as shorter courses of better-tolerated medication, such as 3HP and 4R in contrast to 9H [39, 40]. Providing treatment in locations and via structures that are convenient to patients also reduces barriers, for example in schools [41, 42], in residences [14, 15, 43], or with methadone treatment [16, 44–46]. The convenience of treatment is difficult to separate as a driver of adherence, from the effect of a DOPT strategy. DOT might also be a proxy for frequency of treatment support, as an opportunity for patients to ask questions, report side-effects, and generally engage with providers [47].

In contrast and depending on context and other program characteristics, DOT can also be perceived as punitive and paternalistic and therefore reduce trust and engagement with providers. Patients in high-risk populations find DOT to be humiliating and discriminating [18, 19]. Patients and providers alike acknowledge that the interaction to persuade compliance with DOT is based on authority and subtle threats [18]. Patients with positive experiences have opportunity to negotiate flexibility and had continuity of providers [18].

Our review has several limitations. The wide range of ICERs and of incident cases of active TB indicate uncertain findings. This is reinforced by variation regarding key sensitivity variables, such as expected increase in adherence rates. Several variables likely have local, regional, and cultural differences, for example the already-high baseline adherence rate in Taiwan. Cost of living and costs of medications and materials are higher in remote locations such the Canadian Arctic. Reactivation rates are highly correlated with HIV co-infectivity rates.

There are limitations in using conventional WTP measures. The ICER for 3HP DOT in a low-resource context [33] is above the conventional WTP—three times national GDP—but remains well below the cost of HIV treatment in that context, which is a baseline assumption of that model and an accepted, funded, real-world practice. This contradiction illustrates the utility of reporting relative cost-effectiveness in relation to other, accepted interventions [48]. Further, cost-effectiveness should be accompanied by other considerations in each context such as budget impact, feasibility, transparency, equity, and consistency [49].

From our systematic review, we conclude from the literature that 3HP DOT is cost-effective over the 9H SAT standard at WTP of less than $50,000 per QALY gained in high-income countries.

## Supplementary Information

**Additional file 1.** Search strategy for 3HP cost effectiveness.

**Additional file 2: S1 Table.** Study characteristics.

**Additional file 3: S2 Table.** Key input parameters for modelling studies.

**Additional file 4: S3 Table.** Quality scores based on Drummond checklist.

## Data Availability

All data generated or analysed during this study are included in this published article and in supplementary information files.

## Abbreviations

LTBI: Latent tuberculosis infection
TB: Tuberculosis
9H: 9 months isoniazid
3HP: 3 months weekly isoniazid and rifapentine
4R: 4 months daily rifampicin
DOT: Directly observed treatment
DOPT: Directly observed preventative treatment
CHEERS: Consolidated Health Economic Evaluation Reporting Standards
SAT: Self-administered treatment
ICER: Incremental cost-effectiveness ratio
1HP: 1 month daily isoniazid and rifapentine
USD: United States dollars
CEA: Cost-effectiveness analysis
SAE: Serious adverse effects
WTP: Willingness to pay
