# Supplementary material for: Cost-effectiveness of 3-months isoniazid and rifapentine compared to 9-months isoniazid for latent tuberculosis infection: a systematic review
Source: BMC Public Health. 2022 Dec 7;22:2292. doi: 10.1186/s12889-022-14766-6 (PMC9727859; doi:10.1186/s12889-022-14766-6)
Supplement: Supplementary file 1 — Additional file 1. Search strategy for 3HP cost effectiveness. [file 12889_2022_14766_MOESM1_ESM.docx]

Additional file 1. Search strategy for 3HP cost effectiveness

3 concepts: latent tuberculosis + directly observed therapy + economic/cost

5 databases: PubMed, Embase, CINAHL, Web of Science, LILACS

*PubMed search terms*

- "Latent Tuberculosis"[Mesh] OR Latent Tuberculos*[tw] OR LTBI[tw] or latent TB or latent Mycobacterium tuberculosis[tw] OR latent M. tuberculosis[tw] OR (latent AND tuberculosis)
- "Drug Therapy"[Mesh] OR "drug therapy"[tw] OR chemoprophylaxis[tw] OR pharmacotherap*[tw] OR "Therapeutics"[Mesh] OR therapeutic[tw] OR "directly observed therapy"[tw] OR "directly observed treatment"[tw] OR "DOT"[tw] or "DOPT"[tw]
- "cost-benefit"[tw] OR cost[tw] OR economic[tw] OR "cost effectiveness"[tw] OR "cost-utility"[tw] OR "disability adjusted life year"[tw] OR DALY[tw] OR "quality-adjusted life year"[tw] OR QALY[tw] OR "cost benefit analysis"[tw] OR "cost effectiveness analysis"[tw] OR "quality of life"[tw] OR utility[tw]

*Embase*

- 'latent tuberculosis'/exp OR 'latent tuberculosis' OR 'latent tuberculos*':ab,ti,kw OR 'ltbi':ab,ti,kw OR ('latent':ab,ti,kw AND 'tuberculosis':ab,ti,kw) OR 'latent tb':ab,ti,kw OR 'latent mycobacterium tuberculosis':ab,ti,kw OR 'latent m. tuberculosis':ab,ti,kw
- 'drug therapy'/exp OR 'chemoprophylaxis'/exp OR 'directly observed therapy'/exp OR 'short course therapy'/exp OR 'pharmaceutical care'/exp OR 'drug therap*':ab,ti,kw OR 'DOT':ab,ti,kw OR 'DOPT':ab,ti,kw OR 'pharmacotherap*':ab,ti,kw or 'therapeutic':ab,ti,kw OR 'chemoprophylaxis':ab,ti,kw OR 'directly observed therapy':ab,ti,kw OR 'directly observed treatment':ab,ti,kw
- 'economic evaluation'/exp OR 'economic eval*':ab,ti,kw OR 'cost':ab,ti,kw OR 'economic':ab,ti,kw OR 'cost effectiveness':ab,ti,kw OR 'cost benefit analysis':ab,ti,kw OR 'cost-utility':ab,ti,kw OR 'disability adjusted life year':ab,ti,kw OR 'daly':ab,ti,kw OR 'quality-adjusted life year':ab,ti,kw OR 'qaly':ab,ti,kw OR 'cost effectiveness analysis':ab,ti,kw OR 'quality of life':ab,ti,kw OR 'utility':ab,ti,kw

updated search Embase (change in syntax rules)

- exp 'latent tuberculosis'/ or 'latent tuberculosis'.mp. or 'latent tuberculos*':ab,ti,kw.mp. or 'ltbi':ab,ti,kw.mp. or ('latent':ab,ti,kw and 'tuberculosis':ab,ti,kw).mp. or 'latent tb':ab,ti,kw.mp. or 'latent mycobacterium tuberculosis':ab,ti,kw.mp. or "latent m. tuberculosis:ab,ti,kw".mp
- exp 'drug therapy'/ or exp 'chemoprophylaxis'/ or exp 'directly observed therapy'/ or exp 'short course therapy'/ or exp 'pharmaceutical care'/ or 'drug therap*':ab,ti,kw.mp. or 'DOT':ab,ti,kw.mp. or 'DOPT':ab,ti,kw.mp. or 'pharmacotherap*':ab,ti,kw.mp. or 'therapeutic':ab,ti,kw.mp. or 'chemoprophylaxis':ab,ti,kw.mp. or 'directly observed therapy':ab,ti,kw.mp. or 'directly observed treatment':ab,ti,kw.mp.
- exp 'economic evaluation'/ or 'economic eval*':ab,ti,kw.mp. or 'cost':ab,ti,kw.mp. or 'economic':ab,ti,kw.mp. or 'cost effectiveness':ab,ti,kw.mp. or 'cost benefit analysis':ab,ti,kw.mp. or 'cost-utility':ab,ti,kw.mp. or 'disability adjusted life year':ab,ti,kw.mp. or 'daly':ab,ti,kw.mp. or 'quality-adjusted life year':ab,ti,kw.mp. or 'qaly':ab,ti,kw.mp. or 'cost effectiveness analysis':ab,ti,kw.mp. or 'quality of life':ab,ti,kw.mp. or 'utility':ab,ti,kw.mp.

*CINAHL*

- "latent tuberculos*" OR (MH "Tuberculosis") OR "latent TB" OR "latent M. tuberculosis" OR "latent Mycobacterium tuberculosis" OR ("latent" AND "tuberculosis")
- (MM "Drug Therapy+") OR "drug therap*" OR "pharmacotherap*" OR "chemoprophylaxis" OR “therapeutic” OR "directly observed therapy" OR "directly observed treatment" OR "DOT" OR "DOPT"
- ((((cost-benefit) OR (cost) OR (economic) OR (cost effectiveness) OR (cost-utility) OR (disability adjusted life year) OR DALY OR (quality-adjusted life year) OR QALY OR (cost benefit analysis) OR (cost effectiveness analysis))) OR ((quality of life) OR (utility)))

*Web of Science*

- ALL=("latent tuberculos*" OR LTBI or "latent TB" or "latent M. tuberculosis" or "latent Mycobacterium tuberculosis" or (latent AND tuberculosis))
- ALL= ("drug therapy" OR chemoprophylaxis OR pharmacotherap* OR therapeutic OR "directly observed therapy" OR "directly observed treatment" OR DOT or DOPT)
- ALL=("cost-benefit" OR cost OR economic OR "cost effectiveness" OR "cost-utility" OR "disability adjusted life year" OR DALY OR "quality-adjusted life year" OR QALY OR "cost benefit analysis" OR "cost effectiveness analysis" OR "quality of life" OR utility)

*LILACS*

- "latent tuberculos*" OR "latent TB" OR "latent M. tuberculosis" OR "latent Mycobacterium tuberculosis" OR ("latent" AND "tuberculosis")
- "DRUG THERAPY/HI" or "DRUG THERAPY/IS" or "DRUG THERAPY/MO" or "DRUG THERAPY/MT" or "DRUG THERAPY/NU" or "CHEMOPROFILAXIS" or "CHEMOPROFILAXY" or "CHEMOPROPHILAXIS" or "CHEMOPROPHYLACTIC" or "CHEMOPROPHYLACTICS" or "CHEMOPROPHYLASIS" or "CHEMOPROPHYLAXIS" or "CHEMOPROPHYLAXIS/" or "CHEMOPROPHYLAXIS/AE" or "CHEMOPROPHYLAXIS/CHEMOTHERAPY" or "CHEMOPROPHYLAXIS/CL" or "CHEMOPROPHYLAXIS/IS" or "CHEMOPROPHYLAXIS/MO" or "CHEMOPROPHYLAXIS/MT" or "CHEMOPROPHYLAXIS/SN" or "CHEMOPROPHYLAXIS/ST" or "THERAPEUTIC" or "PHARMACOTHERAPEUTIC" or "PHARMACOTHERAPEUTICAL" or "PHARMACOTHERAPEUTICASPECTS" or "PHARMACOTHERAPEUTICS" or "PHARMACOTHERAPHY" or "PHARMACOTHERAPIE" or "PHARMACOTHERAPIES" or "PHARMACOTHERAPIES/" or "PHARMACOTHERAPIES/AE" or "PHARMACOTHERAPIES/CL" or "PHARMACOTHERAPIES/EC" or "PHARMACOTHERAPIES/ES" or "PHARMACOTHERAPIES/HI" or "PHARMACOTHERAPIES/IS" or "PHARMACOTHERAPIES/MO" or "PHARMACOTHERAPIES/MT" or "PHARMACOTHERAPIES/NU" or "PHARMACOTHERAPIES/PK" or "PHARMACOTHERAPIES/PX" or "PHARMACOTHERAPIES/SN" or "PHARMACOTHERAPIES/ST" or "PHARMACOTHERAPIES/TD" or "PHARMACOTHERAPIES/TO" or "PHARMACOTHERAPIES/TU" or "PHARMACOTHERAPIES/VE" or "PHARMACOTHERAPY" or "PHARMACOTHERAPY-RELATED" or "PHARMACOTHERAPY.." or "PHARMACOTHERAPY/" or "PHARMACOTHERAPY/AE" or "PHARMACOTHERAPY/CL" or "PHARMACOTHERAPY/EC" or "PHARMACOTHERAPY/ELDERLY" or "PHARMACOTHERAPY/ES" or "PHARMACOTHERAPY/HI" or "PHARMACOTHERAPY/IMMUNOTHERAPY" or "PHARMACOTHERAPY/IS" or "PHARMACOTHERAPY/MO" or "PHARMACOTHERAPY/MT" or "PHARMACOTHERAPY/NU" or "PHARMACOTHERAPY/PK" or "PHARMACOTHERAPY/PX" or "PHARMACOTHERAPY/SN" or "PHARMACOTHERAPY/ST" or "PHARMACOTHERAPY/TD" or "PHARMACOTHERAPY/TO" or "PHARMACOTHERAPY/TU" or "PHARMACOTHERAPY/VE" [Words] [Words]
- (cost-benefit) OR (cost) OR (economic) OR (cost effectiveness) OR (cost-utility) OR (disability adjusted life year) OR DALY OR (quality-adjusted life year) OR QALY OR (cost benefit analysis) OR (cost effectiveness analysis) OR (quality of life) OR (utility)
