# Supplementary material for: Cost-effectiveness of 3-months isoniazid and rifapentine compared to 9-months isoniazid for latent tuberculosis infection: a systematic review
Source: BMC Public Health. 2022 Dec 7;22:2292. doi: 10.1186/s12889-022-14766-6 (PMC9727859; doi:10.1186/s12889-022-14766-6)
Supplement: Supplementary file 4 — Additional file 4: S3 Table. Quality scores based on Drummond checklist. [file 12889_2022_14766_MOESM4_ESM.docx]

S3 Table. Quality scores based on Drummond checklist
